# No Value for Routine Chest Radiography in the Work-Up of Early Stage Cervical Cancer Patients

**DOI:** 10.1371/journal.pone.0131899

**Published:** 2015-07-02

**Authors:** Jacob P. Hoogendam, Ronald P. Zweemer, Helena M. Verkooijen, Pim A. de Jong, Maurice A. A. J. van den Bosch, René H. M. Verheijen, Wouter B. Veldhuis

**Affiliations:** 1 Department of Gynaecological Oncology, Oncology Division, University Medical Center Utrecht, Utrecht, The Netherlands; 2 Department of Radiology, Imaging Division, University Medical Center Utrecht, Utrecht, The Netherlands; Georgetown University, UNITED STATES

## Abstract

**Aim:**

Evidence supporting the recommendation to include chest radiography in the work-up of all cervical cancer patients is limited. We investigated the diagnostic value of routine chest radiography in cervical cancer staging.

**Methods:**

All consecutive cervical cancer patients who presented at our tertiary referral center in the Netherlands (January 2006 – September 2013), and for whom ≥6 months follow-up was available, were included. As part of the staging procedure, patients underwent a routine two-directional digital chest radiograph. Findings were compared to a composite reference standard consisting of all imaging studies and histology obtained during the 6 months following radiography.

**Results:**

Of the 402 women who presented with cervical cancer, 288 (71.6%) underwent chest radiography and had ≥6 months follow-up. Early clinical stage (I/II) cervical cancer was present in 244/288 (84.7%) women, while 44 (15.3%) presented with advanced disease (stage III/IV). The chest radiograph of 1 woman – with advanced pre-radiograph stage (IVA) disease – showed findings consistent with pulmonary metastases. Radiographs of 7 other women – 4 early, 3 advanced stage disease – were suspicious for pulmonary metastases which was confirmed by additional imaging in only 1 woman (with pre-radiograph advanced stage (IIIB) disease) and excluded in 6 cases, including all women with early stage disease. In none of the 288 women were thoracic skeletal metastases identified on imaging or during 6 months follow up. Radiography was unremarkable in 76.4% of the study population, and showed findings unrelated to the cervical carcinoma in 21.2%.

**Conclusion:**

Routine chest radiography was of no value for any of the early stage cervical cancer patients presenting at our tertiary center over a period of 7.7 years.

## Introduction

Cervical cancer is the third most common malignancy in women worldwide, with the highest incidence in developing countries [1,2]. The staging system devised by the International Federation for Gynecology and Obstetrics (FIGO) is centered around the gynecologic examination, aided by a limited number of universally available diagnostic tests (including chest radiography) [3,4,5]. Over the past decades, this clinically oriented approach has allowed for globally uniform, inexpensive cervical cancer staging.

Numerous (inter)national cervical cancer guidelines still adhere to these staging principles, and all include routine chest radiography as the primary diagnostic instrument for detection of thoracic metastatic disease [6,7,8,9,10,11,12]. For example, the US guideline advises its use in all patients except for those with microscopic stage IA1-2, wherein it is considered an optional test [7]. However, limited original research exists to support the routine use of chest radiography in the staging work-up of cervical cancer [12]. Indeed, guidelines [6,7,8,9,10,11] are frequently unable to cite specific references, beyond the FIGO expert opinion based recommendation which was the first to endorse radiography use. However, clear disadvantages such as the radiation exposure, patient strain, healthcare costs and the consequences of false-positive findings, justify a critical assessment of this practice.

The primary aim was to investigate the diagnostic yield of routine chest radiography as part of staging of cervical cancer patients in a tertiary referral center in the Netherlands. Specifically, we evaluated the efficiency of the current practice and whether the addition of a chest radiograph results in upstaging to FIGO IVB (i.e. tumor extension beyond the true pelvis). In addition, we will report the rate of coincidental radiographic findings unrelated to cervical carcinoma.

## Methods and Materials

### Design

In this cross-sectional, diagnostic study we included all consecutive patients who presented to our center between January 1^st^ 2006 and September 1^st^ 2013. Inclusion criteria were: 1) histopathological proof of a malignancy primary to the cervix uteri, and 2) staging was performed at our institution. Patients were excluded when they were lost to follow up within the first 6 months, and when chest radiography was absent or performed with a mobile x-ray unit (i.e. bedside examination). No exclusion based on medical history, including prior malignancies, was performed to prevent selecting an abnormally healthy study population.

All procedures in the presented study followed standard clinical care. Authors RZ, RV and WV were the physicians treating the included patients and obtained verbal informed consent for the diagnostic workup (incl. chest radiography) and subsequently documented this in the patient’s medical file. Author JH anonymized the dataset prior to analysis. Patients were not informed that their results would be anonymously used in this analysis. This practice adheres to all applicable Dutch law, specifically the Medical Research Involving Human Subjects Act (WMO). Likewise, institutional review board approval is implicit under Dutch law because only anonymized and already existing (i.e. retrospective) data were used.

### Staging practice

In the Netherlands, all cervical cancer cases are referred to a specialized–tertiary referral–center. At our institution, the standardized cervical cancer staging protocol adheres to national guidelines and consists of a detailed history, full physical and gynecological examination, and chest radiography [10]. In addition, abdominal ultrasound (before 2008) or contrast enhanced pelvic magnetic resonance imaging (from 2008 onwards) are routinely performed to detect ureteric obstruction. An examination under anesthesia is performed when outpatient based (recto)vaginal examination is inadequate for clinical staging.

Histopathological proof of cervical cancer is required by protocol prior to the initiation of therapy. All histological material, including the original samples provided by referring hospitals, is reviewed by an institutional pathologist specialized in gynecological oncology. The work-up findings are presented in a multidisciplinary meeting to reach consensus on the diagnosis, stage and treatment plan.

### Chest radiography

X-ray radiography of the chest was performed on a digital flat panel detector radiography system (DigitalDiagnost, Philips Healthcare, Best, the Netherlands). The x-ray tube potential was maintained at 125 kV while the exposure intensity was automatically optimized per patient (typically: 1–3 mAs). Radiologic technologists followed an institutional protocol which prescribed the methodology for complete depiction of the thoracic cage in the posterior-anterior and lateral plane. During the examination, patients assumed an upright standing position with elevated arms and full inspiration. The upper body was unclothed, including removal of jewelry, and any draping long hair was lifted. Nipple marking was not routinely performed.

Chest radiographs were reviewed by board certified radiologists who had access to all prior radiological examinations available in the Picture Archiving and Communication System (PACS, Sectra AB, Linköping, Sweden). Clinical information indicating the cervical cancer staging purpose was available. When present, the frequency and location of suspected pulmonary or skeletal metastases were registered for each case, as was the incidence of diagnostic findings unrelated to cervical cancer. Additional diagnostic tests performed to confirm the radiographic suspicion of cervical cancer metastases were also scored. Additional tests included computed tomography (CT) of the chest, positron emission tomography (PET)-CT, repeat chest radiography, referral to a pulmonologist and/or targeted histologic sampling. Adverse events hereof were recorded in adherence to the ‘Common Terminology Criteria for Adverse Events’ version 4.03 guideline created by the department of health of the United States (US) government [13].

The reference standard was defined as detection of pulmonary or thoracic skeletal metastasis within 6 months following the staging chest radiograph determined by a composite of chest CT, PET-CT, repeat chest radiography, histopathological (including autopsy) or cytological sampling when available.

### Statistical analysis

Statistical calculations were performed with the ‘Statistical Package for the Social Sciences’ version 20.0.0 (SPSS, International Business Machines, Armonk, United States of America). Summary statistics and (non-)parametric tests were chosen based on the data type and its distribution. Statistical significance was preset at an alpha of <0.05.

## Results

### Study population

**Fig 1:**
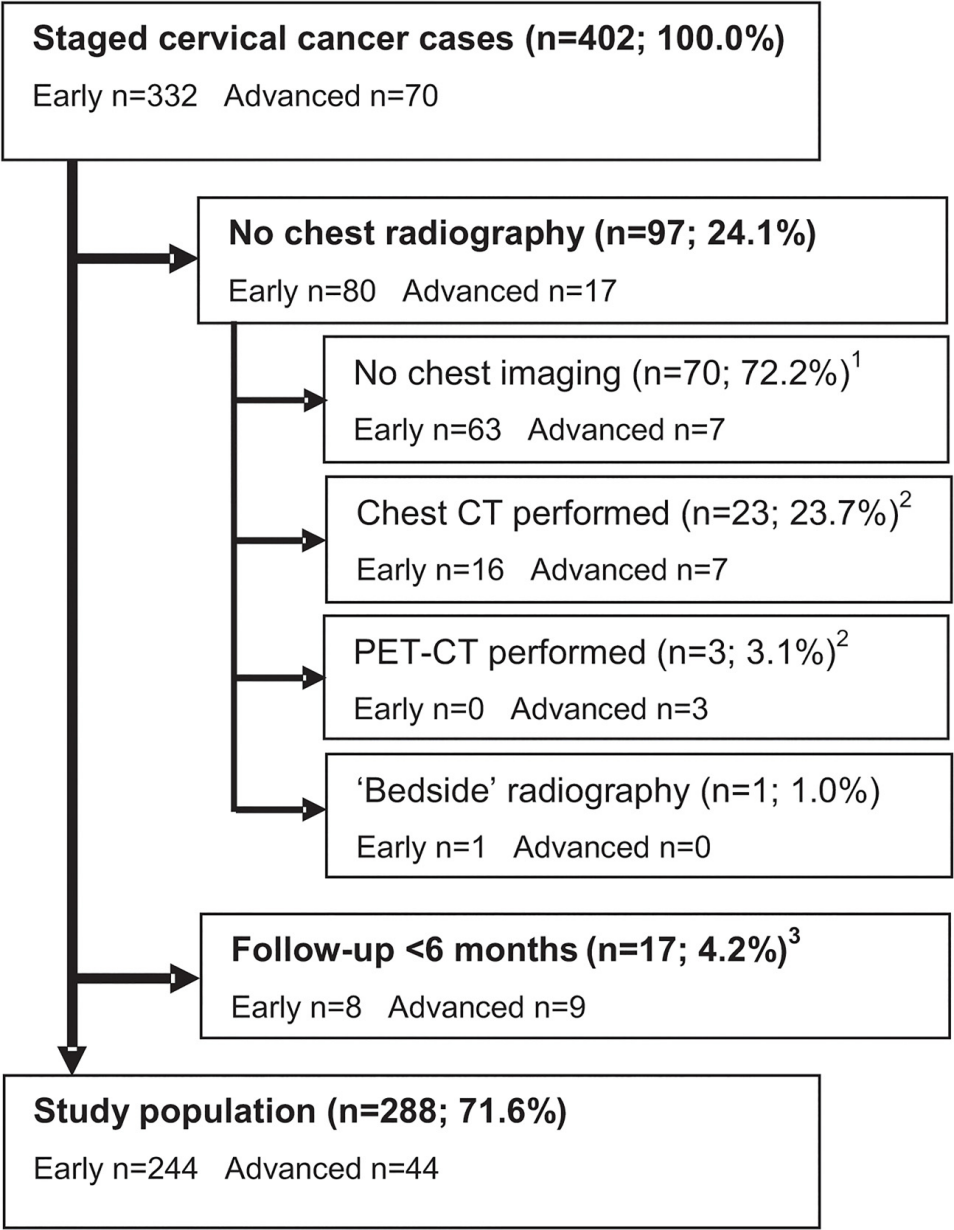
Flowchart displaying the formation of the study population. ^1^ None of these 70 cases had findings during the available follow-up period that would have induced a stage change to IVB. Follow-up of at least 6 months was available in 58/70 (82.9%) cases, including 6 of the 7 advanced cases (85.7%). ^2^ In 20/23 chest CT (87.0%) and all PET-CT cases, imaging was already performed by the referring center. Consequently, chest radiography was not repeated upon formal staging at our institution. In 1 subject (3.8%) pulmonary metastases were found, though in none of the in total 5 FIGO IVB cases (19.2%) upstaging was performed based on chest imaging. ^3^ None of these cases had radiographic findings suspicious for pulmonary or thoracic skeletal metastases. Two patients did have stage IVB cervical cancer, but based on supraclavicular lymph nodal and intrahepatic metastases, not on pulmonary or skeletal metastases that could have been detected on a chest radiograph. CT: Computed tomography; PET: Positron Emission Tomography.

**Fig 2:**
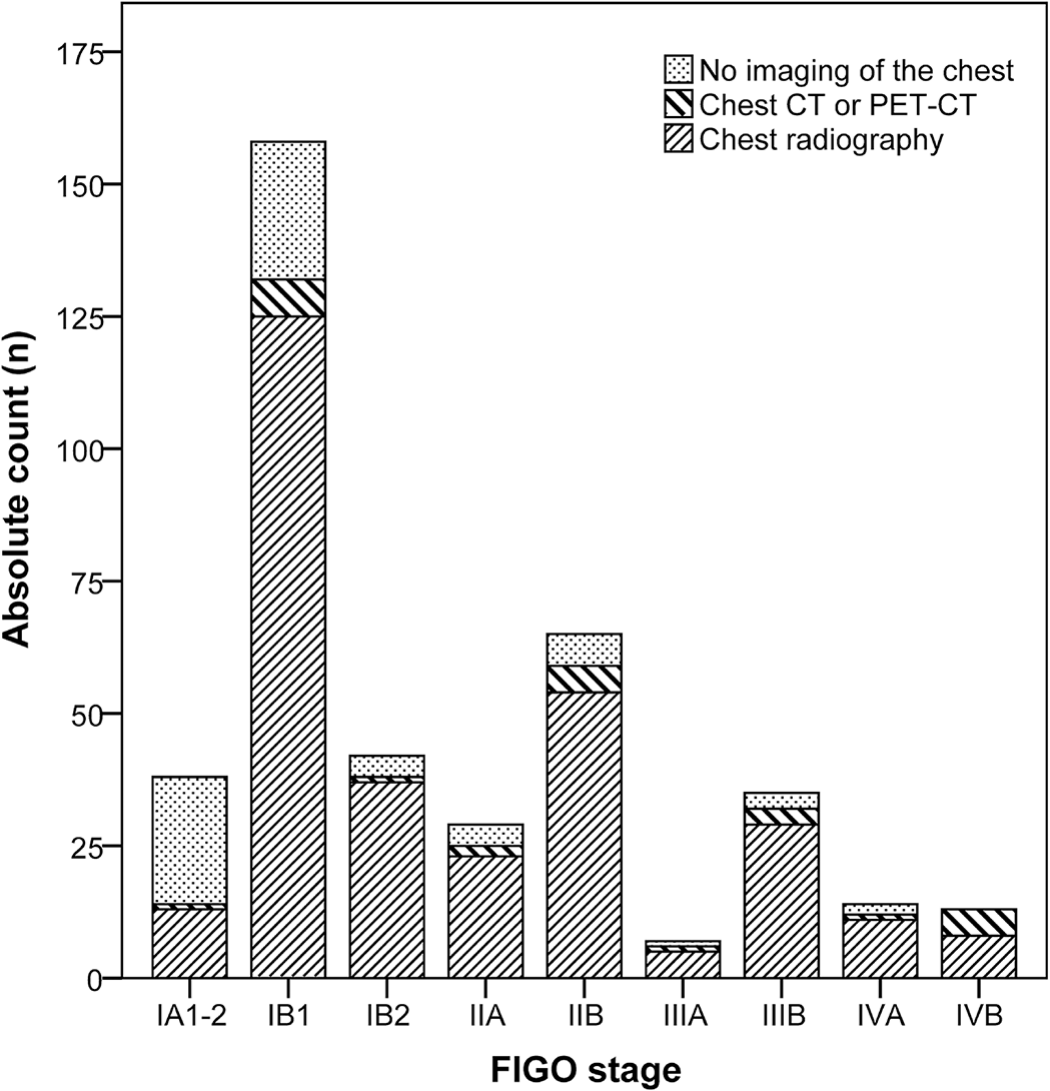
Stacked bar graph of the primary thoracic imaging examinations performed during the staging of all eligible patients (n = 402). CT: Computed Tomography; PET: Positron Emission Tomography; FIGO: International Federation for Gynecology and Obstetrics.

From a total 402 eligible patients, 114 were excluded based on an absent staging chest radiograph (n = 97; 24.1%) or <6 months follow-up (n = 17; 4.2%). Fig 1. None of these excluded lost-to-follow-up cases had radiographic findings suspicious for pulmonary or thoracic skeletal metastases. In the group with no radiograph, 26/97 women had already undergone chest CT (n = 23) or PET-CT (n = 3) in the referring hospital and chest radiography was omitted during formal staging at our institution. This group included a total 5 IVB cases, in all of whom stage IVB cervical cancer was already diagnosed prior to imaging (i.e. no upstaging occurred). A second reason for omitting a chest radiograph–in 24/97 patients–was microscopic, stage IA1-2 cervical cancer. At 63.2%, omission of radiography was much more common in stage IA1-2 patients than in other FIGO stages (p<0.001). The median percentage of cases with no chest imaging in stage IB1 –IVB was 11.7% (range: 0.0 ─ 16.5%), with no statistically significant differences among these stages. Fig 2.

Thus, the study population comprised 288 patients of which 171 (59.4%), 73 (25.3%), 29 (10.1%) and 15 (5.2%) women had stage I, II, III or IV respectively. Table 1. A total of 268 patients (93.1%) were treated with curative intent by either surgery (n = 96; 35.8%), (chemo)radiotherapy (n = 110; 41.0%) or a combination of both (n = 62; 23.1%). Of those, ‘no evidence of disease’ was achieved in 256 (95.5%), of which 35 women (13.7%) had a recurrence after a median of 14 months follow-up (range: 7 ─ 50 months). Hereof, 7 women presented with a recurrence which included pulmonary metastases (median 14 months, range 10 ─ 24 months follow-up) while all had unremarkable chest radiographies at workup. The 5-year overall and disease specific survival rates (n = 288) were 71.9% and 79.2%, respectively.

**Table 1:** Baseline characteristics of the study population (n = 288).

Median age at presentation (range)		46.4 (24.3 ─ 89.8) years	
		**n**	**percentage**
Pulmonary history	Asthma	11	3.8%
	COPD	9	3.1%
	Tuberculosis	1	0.3%
	Other	1	0.3%
Smoking status	Current	94	32.6%
	Stopped	52	18.1%
	Never smoked	134	46.5%
	Unknown	8	2.8%
History of a prior malignancy^1^		10	3.5%
FIGO stage cervical cancer	IA1-2	12	4.2%
	IB1-2	159	55.2%
	IIA1-2	22	7.6%
	IIB1-2	51	17.7%
	IIIA	4	1.4%
	IIIB	25	8.7%
	IVA	9	3.1%
	IVB	6	2.1%
Tumor histology	Squamous cell carcinoma	219	76.0%
	Adenocarcinoma	58	20.1%
	Adenosquamous cell carcinoma	7	2.4%
	Other	4	1.4%
Tumor differentiation grade	I	27	9.4%
	II	163	56.6%
	III	76	26.4%
	Undefined	22	7.6%
Lymph-vascular space invasion		95	33.3%

^1^ Excluding all types of skin cancer except melanoma.

COPD: chronic obstructive pulmonary disease, FIGO: International Federation for Gynecology and Obstetrics.

### Chest radiography

Chest radiography was unremarkable in 220/288 women (76.4%). In one woman (0.3%) radiography showed findings consistent with pulmonary metastases. The radiographs of 7 (2.4%, 95% confidence interval (CI): 1.1–5.2%) other women– 4 early, 3 advanced stage disease–were suspicious for pulmonary metastases. In those 7 cases, 4 chest CT’s, 2 PET-CT’s and 2 repeat chest radiography exams were performed, confirming pulmonary metastases in only 1 case. The remaining 6 cases, including all women with early stage disease, were false-positives (2.1%, 95%CI: 0.8–4.7%) with imaging demonstrating only non-specific benign pulmonary nodules. Two of these women were active smokers and 4 never smoked. Not a single patient was referred for further examination by a pulmonologist. At six month’s follow-up no false-negatives had occurred.

Of the 244 women with pre-radiograph stage I/II disease none showed evidence of pulmonary metastases at initial radiography or during 6 months follow up. Two women with already advanced pre-radiograph stage IIIB and IVA disease were upstaged to stage IVB due to pulmonary metastases. In both, the management plan changed to palliative care. This corresponds to a 4.5% (95%CI: 0.8–16.7%) prevalence of pulmonary metastases in women with pre-radiograph stage III/IV cervical cancer. Table 2.

**Table 2:** Outline of FIGO stage IVB cervical cancer cases in the study population.

	Case description	IVB defining disease site(s)	Pre-radiograph stage	Radiography outcome	Secondary diagnostics^1^
1	49 years, gr 2 SCC, palliative chemotherapy	Supraclavicular LN	IVB	No abnormalities	None
2	65 years, gr 2 SCC, palliative radiotherapy	Lung	IIIB	Solitary lung metastasis	None
3	31 years, gr 2 SCC, palliative chemotherapy	Para-aortal LN, Mediastinal LN, Supraclavicular LN	IVB	No abnormalities	None
4	54 years, gr 2 SCC, experimental therapy (trial)	Liver	IVB	No abnormalities	None
5	48 years, gr 3 SCC, palliative chemoradiotherapy	Lung	IVA	Multiple lung metastases	Chest CT
6	60 years, gr 3 SCC, palliative chemotherapy	Inguinal LN	IVB	Emphysema	None

Cases no. 1 through 5 ultimately died due to cervical cancer while case 6 received palliative care at the conclusion of this study.

^1^ Indicated solely by chest radiography.

Gr: differentiation grade, SCC: squamous cell carcinoma, LN: lymph node(s)

No thoracic skeletal metastases were identified on the staging chest radiography or during the 6 months follow up.

In addition to the false-positive radiographs, in another 61/288 women (21.2%) one or more findings unrelated to cervical cancer were identified. These included thoracic spondylosis (n = 25; 8.7%), cardiomegaly (defined as cardiothoracic ratio ≥0.5) (n = 20; 6.9%), pulmonary emphysema (n = 10; 3.5%), thoracic scoliosis (n = 9; 3.1%), atelectasis (n = 3; 1.0%), old clavicle/rib fractures (n = 2; 0.7%) and various other abnormalities (n = 6; 2.1%). In none of these cases did the radiograph mandate or result in immediate intervention.

## Discussion

Limited original research exists to support the routine use of chest radiography in the staging work-up of cervical cancer. Here, we have analyzed the data from all consecutive cervical cancer patients presented to our tertiary referral center over a 7½ year period.

Routine chest radiography did not identify pulmonary or skeletal metastases in any of 244 patients with stage I/II disease. Consequently, no stage shift occurred. Nor did radiography in these patients yield any secondary health benefits by detection of unrelated pathology. We fear that routine chest radiography exposes early stage cervical cancer patients to ionizing radiation, raises cost-utility concerns and places them at risk for false-positive findings, without a clear benefit in return.

Radiography did lead to upstaging to FIGO stage IVB in 2 patients with already advanced, pre-radiograph stage IIIB and IVA, cervical cancer. This is consistent with the results of Massad et al., in which radiography only identified pulmonary metastases in pre-radiograph stage IIIB patients [14]. In developed countries, these advanced pre-radiograph stage patients are likely to undergo additional cross-sectional imaging, allowing the aforementioned radiograph to still be omitted. However, especially in low resource settings where alternatives are scarce, continued used of radiography in this subgroup of patients can be defended.

Overall, we found a prevalence of pulmonary metastases of 0.7% (2/288). This is in line with the 0.4 ─ 1.6% reported in older studies and supports the generalizability of the findings reported here [15,16,17,18,19,20]. Two studies specified the pre-radiograph stage and both reported pulmonary metastases in 0.6% of early stage cases (disregarding cases with a second primary tumor) and 2.0–2.7% in advanced patients [15,16].

The low pretest probability of finding metastases relative to the high background prevalence of non-specific pulmonary nodules on CT (comparable age group, non-cancer patients: 13 ─ 18% [21,22]), can raise additional uncertainty when cross-sectional imaging is unselectively used. In a minority of our initial patient population chest CT (n = 23) or PET-CT (n = 3) had been performed as the initial thoracic imaging method. Although 1 patient did have pulmonary metastases, in none of the in total 5 FIGO IVB cases did the chest CT or PET-CT findings alter the clinical stage. This is consistent with 4 of the ultimately 6 IVB cases of the radiography population (Table 2: cases 1, 3, 4 and 6). Overall, this reaffirms that thoracic metastatic disease is rarely an isolated reason for upstaging, and even if so, only in cases which have otherwise already been diagnosed with advanced stage disease.

From a statistical standpoint, the absence of any metastases among our ‘sample’ of 244 early stage patients does not necessarily equal a population probability of 0.00%. It is possible, due to random chance, that our series of 244 negative cases was encountered by accident only. However, based on a power of 80% (5% alpha, one sided test), we can state that our sample size is sufficient to exclude that the real population probability lies above 0.63% ('pwr' package version 1.1–2 within R version 3.0.3, R foundation for statistical computing, Vienna, Austria). Furthermore, our significant underrepresentation of IA1-2 patients likely allows for an even lower boundary.

Certain limitations of this study merit further clarification. Firstly, a substantial proportion (28.4%) of eligible patients could not be included, mainly because of absent radiography in low risk patients. We found that stage IA1-2 patients were selectively underrepresented, which likely strengthens rather than refutes our conclusions on radiography’s absent value. Also, as stated earlier, in the subgroup which received immediate cross-sectional imaging instead of a radiograph, no stage changing findings were detected.

Secondly, despite our 7.5 year inclusion interval the population size and especially the number of ‘event’ cases remains limited, which influences precision. This is unfortunately inherent to the rarity of pulmonary and thoracic skeletal metastases in cervical cancer.

Thirdly, the cut-off of 6 months follow-up incorporated into our reference standard can be considered arbitrary. Current literature does not offer any guidance on an optimal length of time for this purpose. Though to minimize the risk of bias in our study, this cut-off was determined before the data-collection was started.

Finally, the radiography data used in our study were generated during the initial clinical review and no ‘expert’ revision was performed. Such a revision could ensure a standardized review and scoring method to minimize–or even exclude when only one expert is used–any inter-observer variability. However, we deliberately chose to follow regular clinical reality to increase the external validity (i.e. generalizability) of our study.

In conclusion, our study showed no value for routine chest radiography in the workup of early stage (stage I/II) cervical cancer patients. In none of these women did the radiograph identify pulmonary metastases or alter the FIGO stage. In advanced cases (stage III/IV), the continued use of routine chest radiography can be considered if cross-sectional imaging is not routinely employed or available. Nevertheless, even in these patients, pulmonary and skeletal metastases remain an infrequent finding at staging and are mostly diagnosed in patients already staged as IVB, making upstaging through radiography (its primary aim) uncommon. While further studies on advanced stage patients are preferred, careful consideration should be given to abandoning the current unselective use of chest radiography in early stage patients.
